# Exploring Psychological Prehabilitation in Complex Abdominal Wall Reconstruction: A Prospective Pilot Study

**DOI:** 10.3389/jaws.2025.15195

**Published:** 2025-09-29

**Authors:** D. L. C. de Jong, J. A. Wegdam, I. C. M. Driessen, Y. van Os, S. W. Nienhuijs, T. S. de Vries Reilingh

**Affiliations:** ^1^ Department of Surgery, Catharina Ziekenhuis, Eindhoven, Netherlands; ^2^ Department of Surgery, Elkerliek Ziekenhuis, Helmond, Netherlands

**Keywords:** psychological, prehabilitation, complex abdominal wall reconstruction, PROM, preoperative optimalization

## Abstract

**Introduction:**

Prehabilitation strategies for patients with complex abdominal wall hernias primarily target physical optimization. However, existing psychological factors like anxiety, depression, and post-traumatic stress disorder are commonly observed in this population. Despite this, they remain underexplored notwithstanding their impact on recovery, complications, and quality of life. This study investigates the prevalence of psychological comorbidities in patients undergoing complex abdominal wall repair (CAWR).

**Methods:**

In this prospective cohort study, consecutive patients planned for CAWR (September 2024-June 2025) were recruited. Complex hernias were predefined. Preoperative assessments focused on the presence of anxiety, depression, post-traumatic stress disorders, self-efficacy and quality of life by validated questionnaires: HADS, Mastery and PCL-5. Associations between psychological factors and postoperative pain scores, opioid use, length of stay and complications were explored.

**Results:**

A total of 16 (46%) out of 35 eligible patients completed all preoperative psychological questionnaires. Sixty-nine percent of patients screened positive for at least one psychological risk factor: sixty percent for anxiety and/or depression, thirteen percent for PTSD and fifty-three percent of patients had low self-efficacy. Median QoL score was 47/120.

**Conclusion:**

This study identified that the threshold to participate in this study was very high. Secondly, two-thirds of patients assessed experienced one or more psychological comorbidities. A relation in these patients with prolonged postoperative pain was observed. These findings suggest that psychological comorbidities may represent a clinically relevant risk factor. Further research is warranted to better understand their role and to evaluate whether targeted psychological prehabilitation could improve outcomes.

## Introduction

Current multimodal approaches of patients with complex abdominal wall hernias predominantly focus on improving the preoperative *physical* condition [1, 2]. Protocolized prehabilitation interventions such as smoking cessation, weight loss programs, or abdominal wall muscle elongation by Botulinum Toxin A (BTA) injection, have clearly demonstrated to reduce postoperative complications [3–8]. However, while many patients with complex abdominal wall hernias have experienced multiple, mostly negative, events in the healthcare system, their neurocognitive and emotional wellbeing may also have been significantly impacted [9]. Any pre-existing anxiety, depression, or other psychological condition will also play a role in outcome after every subsequential surgery.

High levels of preoperative anxiety are associated with heightened sensory sensitivity, lower pain thresholds, and increased postoperative complications and opioid use [10]. In line with this, psychological conditions like depression contribute to delayed recovery, extended hospital stays, and elevated readmission rates [11]. Emerging evidence highlights how psychological comorbidities, including self-efficacy - a person’s belief in their ability to influence their own health–play a crucial role in promoting better health behaviours and overall wellbeing [12].

While addressing a patient’s preoperative *psychological* status has potential to further improve postoperative outcomes, a comprehensive prehabilitation approach addressing both physical and psychological comorbidities by a multidisciplinary team is needed [13]. Although evaluation and optimization of physical health has become a cornerstone of prehabilitation, the preoperative period also offers a unique opportunity to address psychological factors [14, 15].

Incorporation of mental health interventions during the preoperative period to enhance psychological resilience and improve surgical outcomes is termed *psychological prehabilitation* [16]. Psychological prehabilitation has shown promise in other surgical domains, such as oncological and neurosurgical care pathways [17]. Psychological prehabilitation has been shown to reduce stress responses, improve immune function, and enhance postoperative recovery. This includes reduction in length of stay, pain, anxiety, and depression [16, 18]. While complication rates may remain unaffected, previous studies have consistently demonstrated improvements in quality of life (QoL) and psychological resilience after psychological prehabilitation [19, 20].

Patients undergoing complex abdominal wall repair (CAWR) often face complex and prolonged surgical trajectories, characterized by multiple previous operations, longstanding symptoms, and significant impacts on daily activities. Furthermore, trust issues and doubts about recovery, can worsen psychological distress [19, 21, 22]. The high postoperative complication rates associated with CAWR amplify the need for a structured prehabilitation trajectory. Preoperative management of psychological comorbidities could improve patients’ surgical experience and recovery [23].

Despite a lack of specific research on psychological prehabilitation for abdominal wall surgery, exploring the influence of psychological factors on postoperative outcomes, a foundation for the development of evidence-based psychological prehabilitation protocols tailored to CAWR patients is pursued. Utilizing the waitlist to identify psychosocial risk factors can guide personalized care and reduce treatment failure risks [24].

Given the interplay of psychological and physical factors in CAWR patients, and the promising results of psychological prehabilitation in other surgical fields, this pilot study aimed to assess the prevalence of anxiety, depression, and post-traumatic stress symptoms in patients undergoing CAWR.

## Methods

### Design

This study was designed as a prospective cohort study. The primary aim was to explore the presence of psychological comorbidities, anxiety, depression, Post-Traumatic Stress Disorder (PTSD) and low self-efficacy in patients potentially undergoing CAWR. Secondary objectives included the Length of Hospital Stay (LOS), Postoperative Opioid Use, and short-term (≤30 days postoperative) complications.

### Study Population

This study was conducted in an expert centre for CAWR patients. Participants were recruited from the outpatient clinic, between September 2024 and June 2025. Patients were considered eligible if they met the following criteria: adults aged 18 years or older, diagnosis of complex abdominal wall hernia (CAWH) (defect size >10 cm, recurrence, presence of a stoma or infection, or patient-related comorbidities (BMI >30 kg/m^2^, poorly controlled diabetes mellitus, chronic obstructive pulmonary disease requiring inhalation therapy, cardiovascular disease, or immunosuppressive therapy) [25]), and the ability to read and write Dutch. Patients were excluded if urgent or emergency surgical repair was required, in case of pregnancy, or inability to provide informed consent.

Patients received verbal and written information about the study and were given 2 weeks to consider participation, after which informed consent was signed.

### Preoperative Assessment

In case of consent, baseline data were collected, including age, sex, BMI, comorbidities, hernia factors, previous surgeries, and surgical details (duration, open or laparoscopic approach, as well as mesh-type, preoperative BTA administration, Component Separation Technique (CST) used). The Hernia Patient Wound (HPW) classification was assessed for each patient. Additionally, all cases were discussed in an MDT.

Patients who did not complete all three additional preoperative psychological questionnaires were contacted and requested to complete the questionnaires again by email, by phone and in person, during any visit at the outpatient clinic. Only patients who completed the full preoperative screening, including all psychological questionnaires, were included in the analysis of postoperative outcomes.

Furthermore, the HerQLes QoL questionnaire–a hernia-specific QoL questionnaire consisting of 12 items, each scored from 0 to 10 - was filled in, which is standard of care in the recruiting hospital. Non-participating patients received the standard course of treatment.

### Psychological Assessment

Participants were presented three additional validated questionnaires preoperatively.

The first was the *Hospital Anxiety and Depression Scale* (HADS), a 14-item tool designed to assess symptoms of anxiety and depression. It consists of two subscales, one for anxiety (HADS-A) and one for depression (HADS-D), with scores ranging from 0 to 21 for each subscale. A score above 7 on either subscale was used as a cut-off to identify clinically significant symptoms, as it has been widely validated as a threshold indicating moderate levels of anxiety or depression.

The second questionnaire, the *Mastery*, is a 7-item scale measuring self-efficacy, with scores ranging from 7 to 35. A score below 20 was considered indicative of a lower self-efficacy, which has been associated with greater vulnerability to psychological distress. This threshold was selected to identify individuals who may benefit from psychological support.

The third tool used was the *Posttraumatic Stress Disorder Checklist* for DSM-5 (PCL-5), a 20-item questionnaire designed to assess PTSD symptoms based on the DSM-5 criteria. Scores range from 0 to 80, with a score greater than 31 used as a cut-off for clinically significant PTSD symptoms.

These questionnaires were completed prior to any surgical intervention, allowing for the identification of psychological distress early in the preoperative process. The questionnaires are attached in the Appendix.

### Postoperative Assessment

Postoperative outcomes were assessed through the Visual Analog Scale (VAS) for pain on postoperative days 1 and 2, POU calculated as Morphine-Equivalent Dose (MED) mg/kg/12h using an opioid conversion chart, QoL changes measured preoperatively and at 3, 6 and 12 months postoperatively, and postoperative complications within 30 days, classified by the Clavien-Dindo system. As a composite measure of optimal perioperative recovery, Textbook Outcome was assessed, defined as a postoperative hospital stay of less than 5 days without any complications.

### Ethical Considerations

Participation was voluntary, and patients had the opportunity to withdraw at any time without consequences. Evaluation by the medical ethics committee concluded that the study did not fall under the Medical Research Involving Human Subjects Act (Reference number: MECU W.24.094). As a result, ethical approval was not required. This study adhered to the principles of the Declaration of Helsinki.

### Data Analysis

Due to the limited expected sample size, statistical analysis primarily focused on descriptive statistics and effect sizes. This approach aimed to provide preliminary insights into the relationships between psychological factors and postoperative outcomes. Sample size was set at 35 patients, based on the expected number of eligible cases presenting at the outpatient clinic over the inclusion period. Descriptive outcomes are reported using median and interquartile range (IQR), as the data were not assumed to be normally distributed. IQR was preferred over standard deviation or range to provide a more robust summary of central tendency and variability, especially for ordinal data. Primary outcomes included questionnaire completion rates and scores. Secondary analyses explored associations between psychological conditions and postoperative outcomes and pre- to postoperative QoL changes. Given the pilot nature of this study, these outcomes are presented descriptively and no formal comparative analysis was performed.

## Results

**FIGURE 1:**
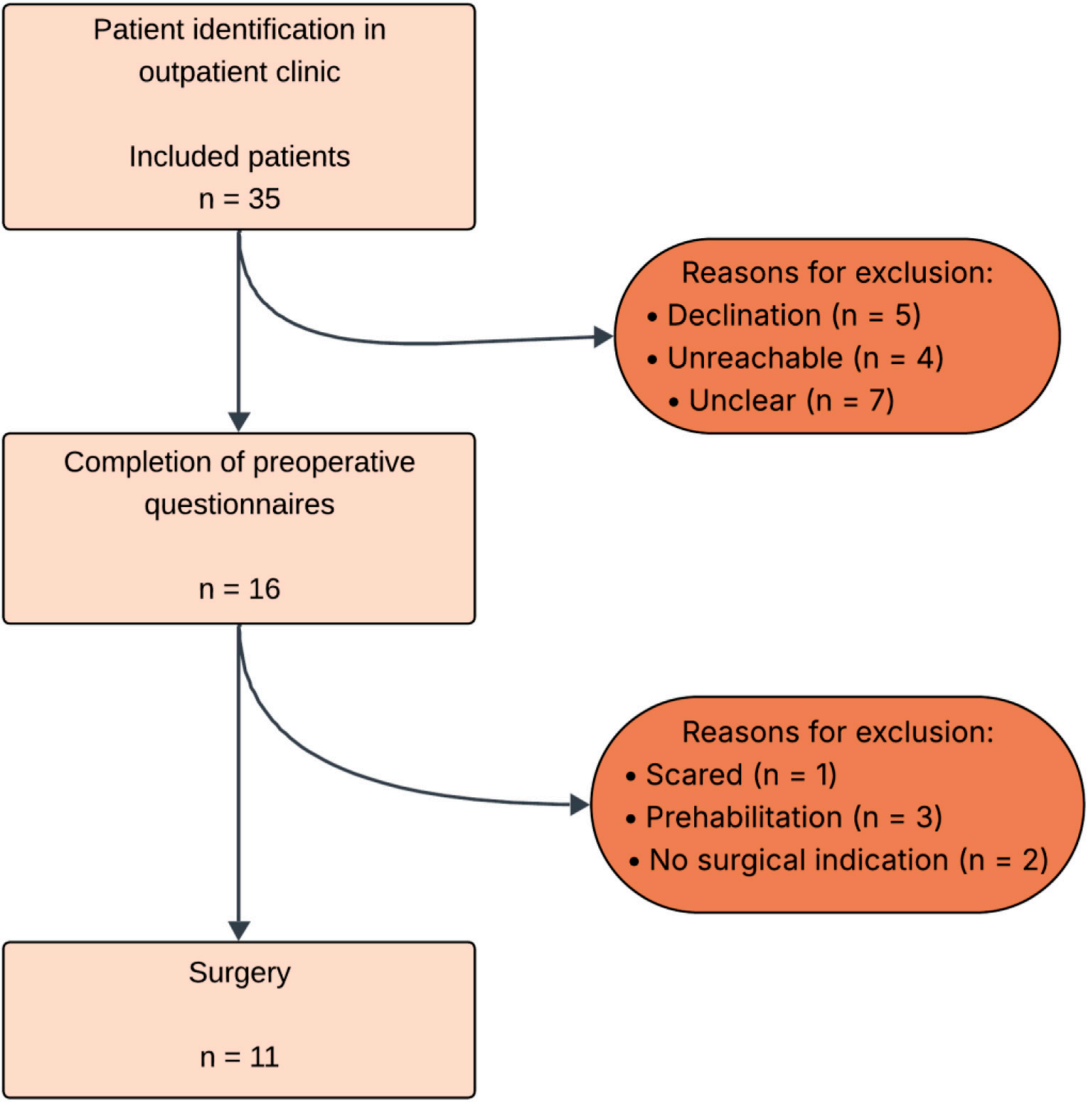
Patient inclusions.

A total of 35 patients were included in the study. All patients provided informed consent to participate with this study. Sixteen patients (46%) completed the full set of the three additional preoperative psychological questionnaires, despite three or more attempts to contact eligible patients again. Five patients explicitly declined participation. Seven patients failed to return questionnaires for unclear reasons despite initial informed consent. The remaining four patients could not be reached (Figure 1).

The baseline characteristics of the participants are presented in Table 1. The mean age of participants was 62.3 years, with equal gender distribution. The mean BMI was 27.8 kg/m2, with 25% of patients classified as obese (BMI >30). Median HPW-stage was 2. Surgical details are provided in Table 1. Not all included patients underwent surgery: only a subset of the cohort proceeded to operative treatment. In some cases, this was due to planned prehabilitation prior to surgery, while in others, the burden of complaints did not outweigh the operative risks.

**TABLE 1:** Baseline characteristics.

n	16	(%)	Mean	SD
Patient				
Female sex	8	(50)		
Age (y)			62.3	±9
BMI (kg/m^2^)			27.8	±4.7
BMI >30	4	(25)		
Active smoker	4	(25)		
Use of immunosuppressives	3	(19)		
Diabetes	2	(13)		
COPD/asthma	2	(13)		
Cardiovascular history	7	(44)		
Hernia				
Width (cm)			8.3	±5.6
Loss of domain >20%	1	(6)		
Wound				
Stoma present	5	(31)		
*CDC classification*				
I	9	(56)		
II	1	(6)		
III	5	(31)		
IV	1	(6)		
*HPW*				
I	3	(19)		
II	7	(44)		
III	6	(38)		
IV	0	(0)		
MDT evaluation				
Green	11	(69)		
Orange	5	(31)		
Red	0	(0)		
Surgery	11	(56)		
OVHR	4	(25)		
CST	7	(44)		
Mesh	7	(44)		
Concomitant procedure	4	(25)		

CDC, centres for disease control classification; HPW, hernia, patient wound; MDT, multidisciplinary team meeting; OVHR, open ventral hernia repair; CST, component separation technique.

The results of the psychological assessments are summarized in Table 2. Of the 16 patients who completed the full psychological screening, eleven (69%) screened positive on at least one of the questionnaires. The HADS revealed a median anxiety score of 3.5 (IQR 0–7.5) and a median depression score of 3.5 (IQR 2–6.5). The Mastery questionnaire indicated reduced self-efficacy in 50% of patients, with a median score of 19.5 (IQR 15–25). PCL-5 screening suggested PTSD-consistent symptoms in 12.5% (median 9, IQR 2–15). Preoperative QoL was scored at a median score of 47 (IQR 38–53). Furthermore, 6 patients (38%) screened positive for more than one psychological comorbidity (Table 3).

**TABLE 2:** Questionnaires.

Questionnaire (n)	Median score	IQR	Above cut-off (%)
HADS (16)	8	3–13	9 (56)
Mastery (16)	19.5	15–25	8 (50)
PCL-5 (16)	9	2–15	2 (13)
Any of the above (16)	-		11 (69)

HADS: hospital anxiety and depression score (cut-off >8), Mastery: self-efficacy (cut-off >20), PCL-5: post-traumatic stress disorder screening tool according to DSM-5 (cut-off <31).

**TABLE 3:** Positive questionnaires.

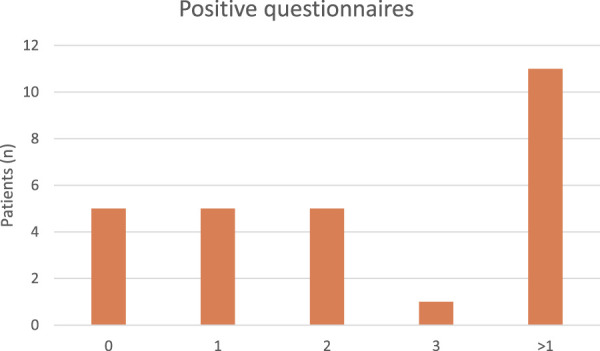

The postoperative outcomes are summarized in Table 4. All patients underwent open ventral hernia repair (OVHR). The median length of hospital stay (LOS) was 5.5 days (IQR 5–7). Postoperative pain, measured using the Visual Analog Scale (VAS), was reported as follow: the median pain score on postoperative day was 5.5 (IQR 2–6), and on day 2, the median score was 2.5 (IQR 0–5). POU was calculated as morphine equivalent X mg/kg/12h, with a median value of 5.6 (IQR 0–15) (Table 5). Within 30 days post-surgery, 36% of patients experienced postoperative complications.

**TABLE 4:** Postoperative outcomes.

n	11	(%)	Median	IQR
VAS POD1			5.5	2–6
VAS POD2			2.5	0–5
Complication	4	(36)		
Length of stay (LOS)			5.5	5–7
LOS >5 days	6	(55)		
Epidural	6	(55)		
Days of epidural			1	0–3
Morphine use (MED)			5.6	0–15
Morphine prescription	3	(16)		
Other analgetics	3	(16)		
Textbook outcome^a^	5	(45)		

^a^ Textbook outcome is defined as LOS <5 days without complications.

LOS, length of stay; MED, morphine equivalent daily dose (mg/12h).

**TABLE 5:** Psychological screening versus postoperative pain.

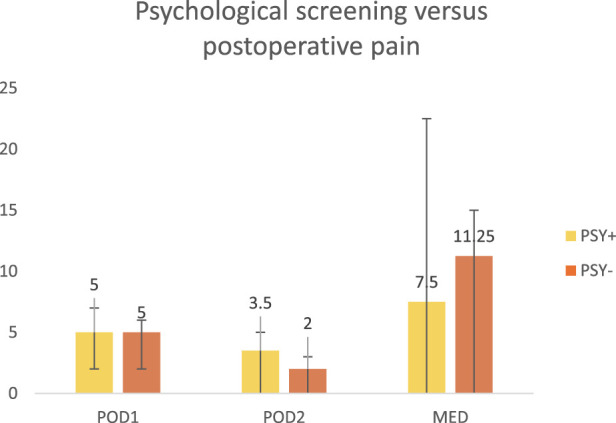

POD1, postoperative day 1; POD2, postoperative day 2; MED, morphine equivalent dose, mg/12h; PSY+, positive screening on psychological questionnaires; PSY -, negative screening on psychological questionnaires.

## Discussion

Although all patients provided informed consent, the response rate was limited, highlighting a potential barrier in feasibility. This underlines the relevance of this pilot phase, which not only aimed to explore the prevalence of psychological risk factors, but also to assess the feasibility and acceptability of administering standardized psychological questionnaires in this patient population. The uptake of PROMs in this cohort was encouraging. Nonetheless, a high prevalence of psychological burden was observed in patients awaiting CAWR. At least 33% of the entire cohort had relevant psychological burden, while among respondents 69% of respondents screened positive for at least one psychological risk factor. Fifty-six percent screened positive for anxiety or depression, and thirteen percent for possible PTSD. Additionally, fifty percent of patients had low Mastery scores, indication limited self-efficacy and coping capability. Given the pilot design and limited sample size, these findings should be interpreted with caution. Still, they support the rationale for further exploration of integrating psychological assessment in surgical pathways, although clinical benefit has not yet been demonstrated.

Psychological comorbidities may represent an additional layer of vulnerability in this population. For example, previous literature shows that smoking and obesity- prevalent in approximately 25%–50% of CAWR patients - are important risk factors for poor surgical outcomes. While our pilot data did not demonstrate a statistically significant correlation between psychological status and surgical outcomes, these findings underscore the need to investigate psychological comorbidity with similar rigour as established physical risk factors in larger-scale studies [1].

The main findings in this study unveil a hidden dimension of patient morbidity. While we previously highlighted the lack of existing literature on this subject, this pilot study represents the first systematic attempt to quantify psychological distress in this surgical population [23]. The holistic approach of this study offers a comprehensive perspective on the interplay between mental health and surgical recovery. This is supported using validated psychological questionnaires, prospective data collection, and the integration of both psychological and physical postoperative outcomes.

Our cohort shows equal or higher psychological burden compared to other surgical fields such as oncological (35%–46%), cardiac (15%–37%), and bariatric surgery (12%–19%), suggesting potential underlying psychological issues [16–18]. Prior studies have identified clear associations between preoperative mental health and postoperative recovery, including reduced pain tolerance, increased opioid consumption, delayed return to function, and impaired immune response [10, 11, 23]. Notably, the prevalence of anxiety and depressive symptoms in our cohort exceeds that of the general population, where estimates range between 10% and 20% depending on age and gender, using the same cut-off values [26]. Our data support the notion that similar mechanisms may be relevant in CAWR.

Psychological distress may influence coping mechanisms, reduce adherence to rehabilitation protocols, and lower motivation to engage in self-care- all of which may contribute to suboptimal surgical outcomes.

Our data suggest that certain subgroups- such as patients with larger hernia width, higher BMI, or multiple previous surgeries- may be particularly vulnerable to psychological burden. This emphasizes the need for individualized preoperative assessments and interventions, ideally embedded in a multidisciplinary care framework. The preoperative period- particularly the waiting list- offers a valuable window for the risk assessment for both physical and psychological risk factors.

Several limitations to our main conclusion should be acknowledged. The small sample size and low response rate of 46% limit the statistical power and generalizability of the study. Low participation may introduce selection bias: psychologically healthy patients might have declined participation, considering the questionnaires irrelevant, while some patients with psychological distress might have avoided participation due to stigma concerns. Moreover, psychological status may itself influence how patients respond to questionnaires, which could distort the true clinical picture. In addition, the burden of completing multiple questionnaires and the need for repeated contact attempts may have further contributed to the limited response rate. Insights into which tools were more burdensome or confusing would inform the design of future studies, however data on this was not available. This reinforces previous literature, that suggests patients facing CAWR often endure longstanding functional impairment, recurrent surgical failures, and substantial disruption in daily life [21, 22]. Additionally, the cross-sectional nature of preoperative assessments provides only a momentary view, as psychological symptoms can fluctuate over time. Postoperative QoL changes are not assessed in this pilot due to the small sample and incomplete follow-up. Similarly, pain outcomes must be interpreted with caution, as we were unable to account for surgical approach, anaesthesia, pre-emptive analgesia, or postoperative pain management protocols, all of which are known to affect pain perception. Future studies could incorporate these factors to explore their potential impact on outcomes. Furthermore, surgical details such as operative approach, mesh type, and preoperative BTA administration were collected but not included in the current analysis, as this study did not aim to perform comparative analyses. Data on wound status was not collected, which could have influenced the choice of surgical technique and may have worsened psychological outcomes in patients with chronic wound issues. Including such data in future studies would provide a more complete understanding of factors affecting both surgical decision-making and PROMs. These limitations indicate the exploratory nature of this pilot study, which cannot yet establish causal associations or determine the prognostic impact of psychological factors on surgical outcomes.

Nevertheless, the findings suggest that psychological comorbidity is a considerable issue in CAWR patients, and advocate for the routine implementation of structured preoperative psychological assessment. At least one-third of the entire patient population experiences relevant psychological symptoms, a prevalence comparable to traditional risk factors such as smoking or obesity. This indicates that psychological distress should be considered a potential risk factor in preoperative risk stratification and management. Future studies should therefore not only optimise feasibility of questionnaire administration (reducing burden, improving timing both pre- and postoperatively, and minimising dropout), but also analyse outcomes in distinct domains—QoL, pain, complications, and LOS—within more homogeneous patient populations and with larger sample sizes to allow meaningful comparisons.

## Data Availability

The raw data supporting the conclusions of this article will be made available by the authors, without undue reservation.

## References

[1] de Jong DLC Wegdam JA Berkvens EBM Nienhuijs SW de Vries Reilingh TS. The Influence of a Multidisciplinary Team Meeting and Prehabilitation on Complex Abdominal Wall Hernia Repair Outcomes. Hernia (2023) 27(3):609–16. 10.1007/s10029-023-02755-6 36787034 PMC9926435

[2] Joslyn NA Esmonde NO Martindale RG Hansen J Khansa I Janis JE. Evidence-Based Strategies for the Prehabilitation of the Abdominal Wall Reconstruction Patient. Plast Reconstr Surg (2018) 142(3 Suppl. l):21s–29s. 10.1097/PRS.0000000000004835 30138261

[3] Jensen KK East B Jisova B Cano ML Cavallaro G Jørgensen LN The European Hernia Society Prehabilitation Project: A Systematic Review of Patient Prehabilitation Prior to Ventral Hernia Surgery. Hernia (2022) 26(3):715–26. 10.1007/s10029-022-02573-2 35212807

[4] de Jong DLC Wegdam JA Van der Wolk S Nienhuijs SW de Vries Reilingh TS. Prevention of Component Separation in Complex Abdominal Wall Surgery by Botox Prehabilitation: A Propensity-Matched Study. Hernia (2024) 28(3):815–21. 10.1007/s10029-023-02929-2 38172376

[5] Timmer AS Claessen JJM Atema JJ Rutten MVH Hompes R Boermeester MA. A Systematic Review and Meta-Analysis of Technical Aspects and Clinical Outcomes of Botulinum Toxin Prior to Abdominal Wall Reconstruction. Hernia (2021) 25(6):1413–25. 10.1007/s10029-021-02499-1 34546475 PMC8613151

[6] Wegdam JA de Vries Reilingh TS Bouvy ND Nienhuijs SW. Prehabilitation of Complex Ventral Hernia Patients with Botulinum: A Systematic Review of the Quantifiable Effects of Botulinum. Hernia (2021) 25(6):1427–42. 10.1007/s10029-020-02333-0 33215244

[7] Levett DZH Grimmett C. Psychological Factors, Prehabilitation and Surgical Outcomes: Evidence and Future Directions. Anaesthesia (2019) 74(Suppl. 1):36–42. 10.1111/anae.14507 30604423

[8] Nathan K Breanna J Robert M. Abdominal Wall Procedures: The Benefits of Prehabilitation. Plast Aesthet Res (2020) 7:7. 10.20517/2347-9264.2019.69

[9] Ramshaw B. Applying Systems and Complexity Science to Real Patient Care. J Eval Clin Pract (2020) 26(5):1559–63. 10.1111/jep.13442 32876990

[10] Hanalis-Miller T Nudelman G Ben-Eliyahu S Jacoby R. The Effect of Pre-Operative Psychological Interventions on Psychological, Physiological, and Immunological Indices in Oncology Patients: A Scoping Review. Front Psychol (2022) 13:839065. 10.3389/fpsyg.2022.839065 35572335 PMC9094613

[11] Neff C Totten C Plymale M Oyler DR Davenport D Roth JS. Associations between Anxiolytic Medications and Ventral Hernia Repair. Hernia (2018) 22(5):753–7. 10.1007/s10029-018-1766-4 29594842

[12] Botha F Dahmann SC. Locus of Control, Self-Control, and Health Outcomes. SSM Popul Health (2024) 25:101566. 10.1016/j.ssmph.2023.101566 38077246 PMC10698268

[13] Parker SG Blake H Zhao S van Dellen J Mohamed S Albadry W An Established Abdominal Wall Multidisciplinary Team Improves Patient Care and Aids Surgical Decision Making with Complex Ventral Hernia Patients. Ann R Coll Surg Engl (2024) 106(1):29–35. 10.1308/rcsann.2022.0167 36927113 PMC10757872

[14] Seiler A Fagundes CP Christian LM. The Impact of Everyday Stressors on the Immune System and Health. In: Choukèr A, editor. Stress Challenges and Immunity in Space: From Mechanisms to Monitoring and Preventive Strategies. Cham: Springer International Publishing (2020). p. 71–92.

[15] Mavros MN Athanasiou S Gkegkes ID Polyzos KA Peppas G Falagas ME. Do Psychological Variables Affect Early Surgical Recovery? PLoS One (2011) 6(5):e20306. 10.1371/journal.pone.0020306 21633506 PMC3102096

[16] Hall AE Nguyen NH Cascavita CT Shariati K Patel AK Chen W The Impact of Psychological Prehabilitation on Surgical Outcomes: A Meta-Analysis and Meta-Regression. Ann Surg (2025) 281:928–41. 10.1097/SLA.0000000000006677 39969855

[17] Tsimopoulou I Pasquali S Howard R Desai A Gourevitch D Tolosa I Psychological Prehabilitation before Cancer Surgery: A Systematic Review. Ann Surg Oncol (2015) 22(13):4117–23. 10.1245/s10434-015-4550-z 25869228

[18] Ramezani A Johnson M Alvani SR Odor A Hosseinpoor S. The P3-Model of Perioperative Psychological Preparation: Pre-Surgical and Pre-Medical Procedural Psychological Preparation and Psychophysiological Interventions. Clin Neurol Neurosurg (2022) 222:107468. 10.1016/j.clineuro.2022.107468 36274352

[19] de Jong DLC Wegdam JA Berkvens EHM de Vries Reilingh TS Nienhuijs SW. Does Quality of Life Improve after Complex Incisional Hernia Repair? A Systematic Review. Hernia (2025) 29(1):110. 10.1007/s10029-025-03300-3 40053186

[20] Philipp M Leuchter M Klar E. Quality of Life after Complex Abdominal Wall Reconstruction. Visc Med (2020) 36(4):326–32. 10.1159/000505247 33005659 PMC7506282

[21] Smith OAM Mierzwinski M McVey J Chitsabesan P Chintapatla S. Abdominal Wall Hernia and Mental Health: Patients Lived Experiences and Implications for Patient Care. Hernia (2023) 27(1):55–62. 10.1007/s10029-022-02699-3 36284067 PMC9595579

[22] Aliotta RE Gatherwright J Krpata D Rosenblatt S Rosen M Gurunluoglu R. Complex Abdominal Wall Reconstruction, Harnessing the Power of a Specialized Multidisciplinary Team to Improve Pain and Quality of Life. Hernia (2019) 23(2):205–15. 10.1007/s10029-019-01916-w 30798398

[23] de Jong DLC de Vries Reilingh TS Nienhuijs SW. Psychological Prehabilitation in Complex Abdominal Wall Reconstruction: Next Level Multidisciplinary Approach? Hernia (2024) 28(3):937–8. 10.1007/s10029-023-02956-z 38190017

[24] Tidmarsh LV Harrison R Finlay KA. Prehabilitation: The Underutilised Weapon for Chronic Pain Management. Br J Pain (2024) 18(4):354–64. 10.1177/20494637241250239 39092207 PMC11289902

[25] Slater NJ Montgomery A Berrevoet F Carbonell AM Chang A Franklin M Criteria for Definition of a Complex Abdominal Wall Hernia. Hernia (2014) 18(1):7–17. 10.1007/s10029-013-1168-6 24150721

[26] Hinz A Brähler E. Normative Values for the Hospital Anxiety and Depression Scale (HADS) in the General German Population. J Psychosom Res (2011) 71(2):74–8. 10.1016/j.jpsychores.2011.01.005 21767686

