# Supplementary material for: Exploring Psychological Prehabilitation in Complex Abdominal Wall Reconstruction: A Prospective Pilot Study
Source: J Abdom Wall Surg. 2025 Sep 29;4:15195. doi: 10.3389/jaws.2025.15195 (PMC12515728; doi:10.3389/jaws.2025.15195)
Supplement: Supplementary file 1 [file Table1.pdf]

PTSD CHECKLIST FOR THE DSM-5

[PTSS Checklist voor de DSM-5 (PCL-5)]

Versie 1.1

Naam: ____________________________ ID-nr: _________________

Studie: ____________________________ Datum:_________________

Disclaimer en Copyright: zie psychotraumadiagnostics.centrum45.nl

Originele tekst: Weathers, Litz, Keane, Palmieri, Marx, & Schnurr - National Center for PTSD (8/14/2013)

Huidige vertaling: Stichting Centrum ’45, Arq Psychotrauma Expert Groep (23/07/2014)

Referentie:

Boeschoten, M.A., Bakker, A., Jongedijk, R.A. & Olff, M. (2014). PTSD Checklist for DSM-5– Nederlandstalige versie. Uitgave: Stichting Centrum ’45, Arq Psychotrauma Expert Groep, Diemen.

Instructies: Hieronder volgt een lijst van problemen die mensen soms kunnen ondervinden na een zeer stressvolle gebeurtenis. Leest u alstublieft elke omschrijving aandachtig door en omcirkel vervolgens één van de cijfers rechts daarvan om aan te geven in hoeverre u er in de afgelopen maand last van heeft gehad.

In hoeverre heeft u in de afgelopen maand last gehad van:	*Helemaal niet*	*Een beetje*	*Matig*	*Nogal veel*	*Extreem veel*
1. Regelmatig terugkerende, onaangename en ongewenste herinneringen aan de stressvolle gebeurtenis?	0	1	2	3	4
2. Regelmatig terugkerende, onaangename dromen over de stressvolle gebeurtenis?	0	1	2	3	4
3. Opeens het gevoel hebben of u gedragen alsof de stressvolle gebeurtenis daadwerkelijk opnieuw plaatsvindt (alsof u terug bent in de tijd dat de gebeurtenis zich afspeelde, en het opnieuw beleeft)?	0	1	2	3	4
4. Erg van streek raken wanneer iets u aan de stressvolle gebeurtenis herinnert?	0	1	2	3	4
5. Een sterke lichamelijke reactie hebben wanneer iets u aan de stressvolle gebeurtenis herinnert (bijvoorbeeld: hartkloppingen, moeite met ademhalen, zweten)?	0	1	2	3	4
6. Het vermijden van herinneringen, gedachten of gevoelens die verband houden met de stressvolle gebeurtenis?	0	1	2	3	4
7. Het vermijden van dingen die herinneringen zouden kunnen oproepen aan de stressvolle gebeurtenis (bijvoorbeeld: bepaalde mensen, plekken, gespreksonderwerpen, activiteiten, voorwerpen of situaties)?	0	1	2	3	4
8. Moeite hebben met het herinneren van belangrijke delen van de stressvolle gebeurtenis?	0	1	2	3	4
9. Sterke, negatieve overtuigingen hebben met betrekking tot uzelf, anderen of de wereld (bijvoorbeeld gedachten hebben zoals: ik ben slecht, er is iets vreselijk mis met mij, niemand is te vertrouwen, de wereld is door en door gevaarlijk?	0	1	2	3	4
10. De schuld geven aan uzelf of aan anderen voor de stressvolle gebeurtenis of de gevolgen daarvan?	0	1	2	3	4
11. Sterke, negatieve gevoelens ervaren zoals angst, afschuw, boosheid, schuld of schaamte?	0	1	2	3	4
12. Verminderde interesse hebben in activiteiten die u eerder graag deed?	0	1	2	3	4
13. Afstand voelen tussen uzelf en andere mensen, of u vervreemd voelen van andere mensen?	0	1	2	3	4
14. Moeite hebben om positieve gevoelens te ervaren (bijvoorbeeld: niet in staat zijn om u gelukkig te voelen of om gevoelens van liefde te hebben voor de mensen die u nabij zijn)?	0	1	2	3	4
15. Prikkelbaarheid, woedeaanvallen, of u agressief gedragen?	0	1	2	3	4
16. Teveel risico's nemen of dingen doen die u schade zouden kunnen toebrengen?	0	1	2	3	4
17. “Superalert”, waakzaam of op uw hoede zijn?	0	1	2	3	4
18. U nerveus voelen of snel schrikken?	0	1	2	3	4
19. Moeite hebben met concentreren?	0	1	2	3	4
20. Moeite hebben met inslapen of doorslapen?	0	1	2	3	4
