# Supplementary material for: Exploring Psychological Prehabilitation in Complex Abdominal Wall Reconstruction: A Prospective Pilot Study
Source: J Abdom Wall Surg. 2025 Sep 29;4:15195. doi: 10.3389/jaws.2025.15195 (PMC12515728; doi:10.3389/jaws.2025.15195)
Supplement: Supplementary file 2 [file Table2.pdf]

Hospital Anxiety and Depression Scale

Wij willen graag weten hoe u zich de laatste tijd heeft gevoeld. Wilt u bij elke vraag het cijfer voor het antwoord dat het meest op u van toepassing is omcirkelen? Denk erom, het gaat bij deze vragen om hoe u zich de laatste tijd (in het bijzonder de afgelopen 4 weken) voelde, dus niet om hoe u zich in het verleden heeft gevoeld. Denk niet te lang na, uw eerste reactie is waarschijnlijk de meest nauwkeurige.

1. Ik voel me de laatste tijd gespannen	3 – meestal<br>2 – vaak<br>1 – af en toe, soms<br>0 – helemaal niet
2. Ik geniet nog steeds van de dingen waar ik vroeger van genoot.	0 – zeker zo veel<br>1 – wat minder<br>2 – duidelijk minder<br>3 – nauwelijks nog
3. Ik krijg de laatste tijd het angstige gevoel alsof er elk moment iets vreselijks zal gebeuren	3 – heel zeker en vrij erg<br>2 – ja, maar niet zo erg<br>1 – een beetje, maar ik maak me er geen zorgen over<br>0 – helemaal niet
4. Ik kan lachen en de dingen van de vrolijke kant zien.	0 – net zoveel als vroeger<br>1 – nu wat minder<br>2 – nu duidelijk minder<br>3 – helemaal niet meer
5. Ik maak me de laatste tijd ongerust.	3 – heel erg vaak<br>2 – vaak<br>1 – niet zo vaak<br>0 – heel soms
6. Ik voel me de laatste tijd opgewekt:	3 – helemaal niet<br>2 – niet vaak<br>1 – soms<br>0 – meestal
7. Ik kan de laatste tijd rustig zitten en me ontspannen:	0 – zeker<br>1 – meestal<br>2 – niet vaak<br>3 – helemaal niet

8. Ik voel me de laatste tijd alsof alles moeizamer gaat.	3 – bijna altijd<br>2 – heel vaak<br>1 – soms<br>0 – helemaal niet
9. Ik krijg de laatste tijd een soort benauwd, gespannen gevoel in mijn maag.	0 – helemaal niet<br>1 – soms<br>2 – vrij vaak<br>3 – heel vaak
10. Ik heb de laatste tijd geen interesse meer in mijn uiterlijk.	3 – zeker<br>2 – niet meer zoveel als ik zou moeten<br>1 – mogelijk wat minder<br>0 – evenveel interesse als voorheen
11. Ik voel me de laatste tijd rusteloos.	3 – heel erg<br>2 – tamelijk veel<br>1 – niet erg veel<br>0 – helemaal niet
12. Ik verheug me van tevoren al op dingen.	0 – net zoveel als vroeger<br>1 – een beetje minder dan vroeger<br>2 – zeker minder dan vroeger<br>3 – bijna nooit
13. Ik krijg de laatste tijd plotseling gevoelens van angst of paniek.	3 – zeer vaak<br>2 – tamelijk vaak<br>1 – niet erg vaak<br>0 – helemaal niet
14. Ik kan van een goed boek genieten of een radio- of televisieprogramma.	0 – vaak<br>1 – soms<br>2 – niet vaak<br>3 – heel zelden

De formulering van de vragen en antwoordmogelijkheden is voor 6 items positief (0-3), voor 8 items negatief (3-0).

Uitleg puntentelling HADS score

De oneven vragen (1, 3, 5, 7, 9,11, 13) hebben betrekking op ANGST → **Totale score** = __________

De even vragen (2, 4, 6, 8, 10, 12, 14) hebben betrekking op DEPRESSIE → **Totale score** = __________

Bij een score op de subschaal van:
0-7 : geen angststoornis of depressie
8-10 : een *mogelijke* angststoornis of depressie
11-21: een *vermoedelijke* angststoornis of depressie
