# Supplementary material for: Exploring Psychological Prehabilitation in Complex Abdominal Wall Reconstruction: A Prospective Pilot Study
Source: J Abdom Wall Surg. 2025 Sep 29;4:15195. doi: 10.3389/jaws.2025.15195 (PMC12515728; doi:10.3389/jaws.2025.15195)
Supplement: Supplementary file 3 [file Table3.docx]

Mastery↓

| **19**. In de volgende lijst staan een aantal beweringen die op uzelf betrekking hebben. Wilt u aangeven in hoeverre u het met de beweringen eens of oneens bent. Wilt u een kruisje zetten achter het antwoord dat het meest voor u van toepassing is. |
| --- |
| **Ik heb weinig controle over de dingen die me overkomen** |
| helemaal mee eens |
| mee eens |
| niet mee eens/ niet mee oneens |
| mee oneens |
| helemaal mee oneens |
| **Sommige van mijn problemen kan ik met geen mogelijkheid oplossen** |
| helemaal mee eens |
| mee eens |
| niet mee eens/ niet mee oneens |
| mee oneens |
| helemaal mee oneens |
| **Er is weinig dat ik kan doen om belangrijke dingen in mijn leven te veranderen** |
| helemaal mee eens |
| mee eens |
| niet mee eens/ niet mee oneens |
| mee oneens |
| helemaal mee oneens |
| **Ik voel me vaak hulpeloos bij het omgaan met de problemen van het leven** |
| helemaal mee eens |
| mee eens |
| niet mee eens/ niet mee oneens |
| mee oneens |
| helemaal mee oneens |
| **Soms voel ik dat een speelbal van het leven ben** |
| helemaal mee eens |
| mee eens |
| niet mee eens/ niet mee oneens |
| mee oneens |
| helemaal mee oneens |
| **Wat er in de toekomst met me gebeurt hangt voor het grootste deel van mezelf af** |
| helemaal mee eens |
| mee eens |
| niet mee eens/ niet mee oneens |
| mee oneens |
| helemaal mee oneens |
| **Ik kan ongeveer alles als ik mijn zinnen erop gezet heb** |
| helemaal mee eens |
| mee eens |
| niet mee eens/ niet mee oneens |
| mee oneens |
| helemaal mee oneens |
